# What Hinders the Development of Green Building? An Investigation of China

**DOI:** 10.3390/ijerph16173140

**Published:** 2019-08-28

**Authors:** Zezhou Wu, Mingyang Jiang, Yuzhu Cai, Hao Wang, Shenghan Li

**Affiliations:** 1 Department of Construction Management and Real Estate, College of Civil and Transportation Engineering, Shenzhen University, Shenzhen 518060, China; 2 Sino-Australia Joint Research Centre in BIM and Smart Construction, Shenzhen University, Shenzhen 518060, China; 3 School of Management Science and Engineering, Central University of Finance and Economics, Beijing 100081, China

**Keywords:** green building development, barriers, PLS-SEM, China

## Abstract

With the rapid development of the economy, people are paying more and more attention to the environmental problems. In this circumstance, the concept of a circular economy is proposed for making efficient use of resources and minimizing the production of waste and other emissions. Each year, the construction sector consumes a vast volume of resources and makes impacts on the environment. To align with the development of the circular economy, the concept of green building is proposed. In China, though the concept of green building has been promoted for decades, the development status is far from optimistic. Thus, this paper aims to investigate the barriers that hinder green building development (GBD) in China. Through a systematic review and semi-structured interviews with experienced industrial practitioners, 24 potential barriers of GBD in China were identified. A questionnaire survey was then conducted for data collection. After descriptive and inferential statistical analyses, a partial least squares structural equation model (PLS-SEM) was established to investigate the impacts of different barriers on GBD. Results showed that the lack of policy and industry guidance, the immature market environment, and the lack of environmental awareness are the most important GBD barriers in China. This research can assist stakeholders in better understanding the status of GBD in China and enable decision-makers to formulate appropriate strategies to promote green building.

## 1. Introduction

With the rapid development of the global economy, environmental problems are becoming more and more prominent [1]. The construction activities consume huge natural resources and generate a large amount of waste and carbon emissions [2,3,4]. According to the statistics, 40% of the global energy consumption is related to buildings, while 50% of global greenhouse gas emissions come from buildings [5]. In order to reduce negative impacts, the philosophy of the circular economy has been emphasized in the construction industry to contribute to sustainable development. Green building is a good example of implementing the principles of the circular economy. For example, Leising et al. [6] investigated the circular economy in the building sector and analyzed three cases which obtained BREEAM (a green building rating tool) certificates. 

Green building is an architectural concept which encourages the use of more environmentally friendly materials and adopts technology for saving resources and reducing waste consumption [7]. To facilitate green building design, several green building evaluation systems which emphasize different aspects of environment (e.g., materials, energy, and water) have been established, such as LEED, BREEAM, Green Mark, Green Star, HK-BEAM [8,9,10,11,12]. In addition, incentives have also been proposed for promoting green building development (GBD), such as tax incentives, subsidies and credit incentives [13]. With the development of the last several decades, there have been some evolutions in the green building field, such as the integration of sustainable initiatives at different urban scales [14], regenerative design [15], and zero urban heat island impact building [16].

In China, the concept of green building has been used in the construction industry for decades [17]. In June 2006, the Ministry of Housing and Urban–Rural Development (MHURD) issued the first national green building evaluation standard: “Evaluation Standard for Green Building (ESGB)” (GB/T50378-2006) [18]. This standard was then updated as the “Assessment standard for green building” (GB/T50378-2014). According to the research conducted by Mao et al. [19], there have already been more than 70 green building evaluation standards which were initiated according to provincial, city and local situations . In some developed cities, such as Shenzhen, it is a mandatory requirement that the newly built buildings should obtain ESGB certificates [20]. With these efforts, it is expected that the total area of green buildings in China will reach two billion square meters by 2020 [21].

According to the above statistics, it seems that green building development in China is under fast development. However, there are still many problems that actually hinder the development of green building. Li et al. [22] claimed that there is not a complete technology system for green building construction. Zhang et al. [23] revealed that the proportions of top-rated and operation-certified housing were only 14.9% and 4.5%, respectively. Liu and Hu [24] argued that the green building market has not been maturely established because the public’s attention toward green buildings are not clear. Currently, it has been revealed that the promotion of green building is still by means of government intervention, and active market participation is insufficient [25,26,27,28]. Thus, it is necessary to identify the barriers that hinder the development of green building in China.

The structure of this paper is as follows. Section 2 describes the research methods used in this study and elaborates on the process of questionnaire design, data collection and data analysis. Section 3 presents the descriptive and inferential statistical analysis results and the implementation of a partial least squares structural equation model (PLS-SEM). Section 4 gives a discussion based on the survey results, and finally, the paper ends with a conclusion section.

## 2. Research Methodology

### 2.1. Identification of Potential Barriers

The potential barriers of green building development were identified from the existing literature. In the current literature, there have already been a number of studies focusing green building development barriers in different countries or regions. For example, Love et al. [29] pointed out that the lack of government incentives and relevant knowledge restricted green buildings in Australia. Williams and Dair [30] argued that the lack of consideration, high perceived costs, and inadequate expertise hindered green building development in England. Lam Patrick et al. [31] conducted a survey of stakeholders in Hong Kong and identified key barriers against green specifications, including additional cost, delay and the limited availability of reliable suppliers. In developing countries, the influencing factors were also investigated. For example, Masrom et al. [32] found that the higher cost and lack of green consciousness restricted sustainable refurbishment of commercial buildings in Malaysia. Chan et al. [33] revealed that the barriers of green building development in Ghana can be grouped into five categories, and the most dominant group was government-related barriers. In China, Zhang et al. [34] investigated the barriers of green property development and found that the higher costs hindered the application of green technologies.

From the above literature, it can be seen that the barriers to green building in each country or region have certain commonalities but also have unique characteristics. Due to the different national conditions, policies and economic levels, people’s awareness of environmental protection is also different. In order to fully address the potential barriers in China, interviews with two experienced industrial professionals were further conducted to confirm the identified barriers. Finally, 24 potential barriers to GBD were confirmed and included in the questionnaire, as shown in Table 1.

### 2.2. Questionnaire Design and Data Collection

Questionnaire surveys were implemented in this study. This method was adopted because it can accurately and concretely measure the process of social activities [35,36,37]. The developed questionnaire was divided into two parts. The first part aimed to collect the background information of the interviewees, including their occupation, working experience, and highest education. The second part invited the respondents to evaluate the identified 24 barriers. A five-point Likert scale was utilized to measure the respondents’ perceptions on the potential barriers. The five-point Likert scale was selected because it is easier for the respondents to express their opinions [38]. In the measurement scale, “1” referred to “Extremely not important” while “5” denoted “Very important”. The scores “2”, “3”, and “4” represented “Not important”, “Neutral”, and “Important”, respectively. The questionnaire also collected respondents’ perception of the overall situation of GBD in China. 

The questionnaire responses were collected with two steps. In the first step, the questionnaire was published online, and the website was distributed in different construction related professional forums. However, only 26 responses were collected. Then, a “snowball sampling” strategy was employed. The questionnaire was sent to the identified professionals by emails and they were invited to invite their colleagues to fill out the questionnaire. Finally, a total of 78 responses were collected.

### 2.3. Data Analysis

The data analysis involved two steps. The first step was descriptive and inferential analyses using the Statistical Package for Social Science (SPSS) 22.0. The SPSS was recognized as one of the most widely used computer comprehensive data processing software (data management, statistical analysis, chart analysis, output management and other functions) in the world [39,40,41]. Descriptive analysis includes the determination of central tendency (mean) and variability (standard deviation) [42]. Inferential analysis includes a one-way analysis of variance (ANOVA) test and exploratory factor analysis (EFA). An ANOVA was used to test whether different groups of respondents had different perceptions on GBD barriers [43], to test which EFA could reduce the number of GBD barrier factors, and to determine the set of manageable factors [44]. The EFA can be used as a precursor for a confirmatory factor analysis (CFA) [45]. The principal components analysis was used in the factor analysis of this study, and Varimax rotation was used as the rotation method [46]. The Kaiser–Meyer–Olkin (KMO) and Bartlett sphericity tests were employed to evaluate the applicability of the factor analysis. Generally, the KMO coefficient was required to be above 0.7, and the Bartlett’s test of sphericity was used to examine whether the correlation matrix was significantly different from zero [47].

The second step was to establish a structural equation model (SEM), which is considered to be one of the most suitable techniques for analyzing variables [48]. The SEM supports the use of multiple predicative variables and allows for measurement errors in independent and dependent variables [49]. The SEM can be divided into two categories: The covariance-based SEM (CB-SEM) and the partial least square SEM (PLS-SEM) [50]. Wei et al. [51] stated that the CB-SEM can minimize the divergence between the estimated and sample covariance matrices, while the PLS-SEM estimates partial model relationships in an iterative sequence of ordinary least squares regressions. Hussain et al. [52] identified the PLS-SEM as one of the most suitable techniques for analyzing variables. Moreover, the PLS-SEM has attracted more and more attention in the field of construction management [53,54]. This is because the data distribution measured in the Likert scale is unknown, and its normality cannot be proven. One of the advantages is that it does not assume the distribution form of any measurement variable [55]. In addition, the PLS-SEM can solve complex problems without requiring large numbers of samples [56,57]. Therefore, this study adopted the PLS-SEM, and the SmartPLS 3.2.8 software was employed. The first step was to conduct a CFA. After checking the reliability and validity of the measurement model, path analysis was conducted to evaluate the relations among the eight factors’ groupings [58]. The CFA can detect relationship between measurement items and their structures [59]. Composite reliability (CR) and Cronbach’s alpha coefficient were used to evaluate internal consistency reliability [60]. Once reliability was evaluated, validity (including convergent and discriminant validity of the structure) must also be evaluated [20]. The cross loads of the measured items were tested to verify discriminant validity. Finally, the PLS-bootstrapping technique was used to estimate the path coefficients [61].

## 3. Results

### 3.1. Descriptive Statistics

The descriptive statistics of the 78 respondents were analyzed using the SPSS software package, and the results are shown in Table 2. From Table 2, it can be seen, among all respondents, scholars accounted for the highest proportion of 35.90%, followed by developers (26.92%), contractors (20.51%) and government employees (16.67%). In addition, most respondents had relatively little work experience, with a total of 92.30% having less than 10 years of work experience. More than 97% of respondents had a bachelor’s degree or above. Furthermore, the questionnaire also counted the number of on-going project workers. The results showed that nearly 40% of the projects had fewer than 50 workers, more than 60% had fewer than 100 workers, and 23.08% were more than 200.

The ranking of the potential barriers was implemented by using the mean and standard deviation of the variables. The variables with higher means were ranked higher. In the circumstance that two variables shared the same mean value, the variables with smaller standard deviation were ranked higher. The ranking of the potential barriers is illustrated in Table 3. From Table 3, it can be seen that the three most critical barriers identified by respondents were B04 (lack of effective supervision from government), B17 (lack of environmental awareness from developer), and B15 (immature green material market). Therefore, respondents mainly focused on policy, awareness and market (the only three barriers with mean values above 4.00).

### 3.2. Analysis of Variance

In order to test whether the respondents have different perceptions based on different backgrounds, an analysis of variance (ANOVA) was conducted. The results from the ANOVA are presented in Table 4. Normally, it is suggested that there is high degree of difference between the tested groups if the *p*-value of a variable is less than 0.05 [72]. In Table 4, F represents the group square difference value of F test, and Sig. represents the test value with significant difference.

From this table, it can be seen that there were three variables with *p*-value less than 0.05 in the “Workplace” group, namely B12, B17, and B21, indicating that the respondents from different affiliations had different perceptions towards these three variables. In terms of the “experience” group, there were also three variables with a *p*-value less than 0.05, namely B10, B16, and B24. For the “gender” group, the variables with *p*-value less than 0.05 were B14, B17, and B23. In the aspect of “education”, four variables had a *p*-value less than 0.05—B3, B5, B16, and B17. With regard to the “number of workers” group, only the variable of B24 shared different perceptions between different groups. Therefore, although the backgrounds of the interviewees were different, they had a good consensus on the cognition of most barriers, which is consistent with Chan, Darko, Ameyaw Ernest and Owusu-Manu [70].

### 3.3. Exploratory Factor Analysis

An exploratory factor analysis (EFA) was conducted for twenty-four barriers in the total sample (Table 1) to reduce the dimension of the factors and to make the classification of ethnic groups manageable. In general, the EFA includes five steps: Research problem design, the construction of correlation matrix, the determination of number of factors to be extracted, factor rotation, and the interpretation of factors [73]. The EFA generally requires a sample size of at least 100; however, if the respondents are experienced professionals, the response number may be less than 100 [71]. The extraction method used in the factor analysis was principle component analysis (PCA), and the rotation method selected was Varimax [74]. The PCA was selected because it could ensure the minimum loss of original information and study the underlying structure of the dataset [75].

The factor analysis result of the selected potential barriers is presented in Table 5.

According to the results of the EFA, the loading of four factors (B09, B10, B11, B16) was lower than 0.500. Liu et al. [76] claimed that the variables with a factor loading less than 0.50 should be deleted, and they were thus excluded from the final frame. Therefore, the remaining 20 potential barriers were extracted from five components, and the total explanatory variance was 66.057%, higher than the general threshold of 60% in social science research [77]. In addition, all the parameter statistics were acceptable, which proves the rationality of the EFA.

The first component consisted of variables B07 (incendiary of construction cost), B13 (economic benefit is not obvious in a short term), B08 (extension of construction period), B12 (higher price of green building), and B14 (lack of financial support). Most of the variables in this component are related to economic feasibility and efficiency, so this component can be named as “economic efficiency (EE)”, and the five variables of this component can be renumbered as EE1, EE2, EE3, EE4, and EE5, in turn.

The second component contained variables B22 (lack of green construction training), B23 (lack of publicity for green buildings), B21 (ineffectiveness of demonstration green building), B24 (limited benefit to enterprise reputation), and B15 (immature green material market). Most of the variables in this component are related to the current market situation and industry environment, so this component can be called “market environment (ME)”, and the five variables of this component can be renumbered as ME1, ME2, ME3, ME4, and ME5, in turn.

The third component contained variables B18 (lack of environmental awareness from contractor), B17 (lack of environmental awareness from developer), B19 (lack of environmental awareness from public) and B20 (low demand for green buildings). Most of the variables of this component are related to the environmental protection awareness of different stakeholders, so this component can be called “stakeholder awareness (SA)”, and the four variables of this component can be renumbered as SA1, SA2, SA3, and SA4, in turn.

The fourth component contained variables B04 (lack of effective supervision from government), B02 (lack of industrial guidance), and B01 (lack of regulations and policy). The variables of this component are mostly related to industry regulations and related guidance, so this component can be called “industry policy (IP)”, and the three variables of this component can be renumbered as IP1, IP2, and IP3, in turn.

The fifth component contained variables B05 (inadequate support from related green building institutions), B06 (low level of green design), and B03 (lack of effective green building development modes). The variables of this component are mostly related to technical feasibility and development mode, so this component can be called “technical support (TS)”, and the three variables of this component can be renumbered as TS1, TS2, and TS3, in turn.

According to the above analysis, it can be concluded that the current GBD barriers in China mainly include the following five aspects: Low economic efficiency, poor market environment, weak environmental awareness of stakeholders, imperfect industrial policies, and lack of relevant technical support.

### 3.4. Partial Least Square Structural Equation Model (PLS-SEM)

**Figure 1:**
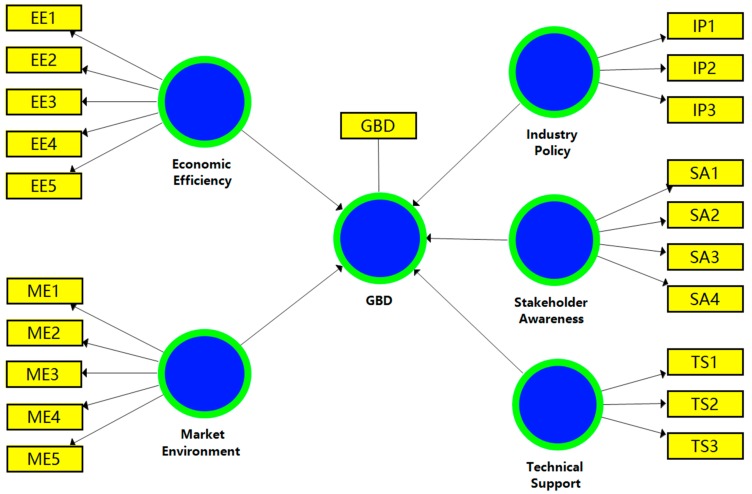
The initial partial least squares structural equation model (PLS-SEM).

In order to establish a hypothetical partial least squares structural equation model (PLS-SEM), SmartPLS 3.2.8 software was used to perform measurement and structural model evaluation to analyze the impact of different components on GBD. The initial PLS-SEM is shown in Figure 1. The measurement items and structural models used in the figure are listed in Table 5. The two-step method recommended by C. Anderson and Gerbing [78] was adopted to analyze and interpret the results of the PLS-SEM: (1) The assessment of the outer measurement model; (2) the evaluation of the inner structure model.

#### 3.4.1. Assessment of Outer Measurement Model

Wen and Li [79] suggested that the reliability and validity of internal consistency should be evaluated when evaluating a measurement model. Cronbach’s α and composite reliability (CR) are the evaluation indexes for internal consistency to determine the adequacy of a measurement model. Nunnally and Bernstein [80] suggested that the values of Cronbach’s α and CR should both exceed the threshold of 0.700, indicating a high internal consistency. In addition, convergent validity was evaluated of average variance extracted (AVE) values. Fornell and Larcker [81] suggested that 0.5 should be the critical criterion for AVE. In order to achieve a satisfactory level of convergence validity, the factor load of each measurement item needs to exceed 0.500 [82]. The assessment results of reliability analysis and convergent validity are shown in Table 6. As can be seen from Table 6, the range values of all factor loads were between 0.697 and 0.902, Cronbach’s α values were between 0.741 and 0.863, AVE values were between 0.617 and 0.715, and CR values were all greater than 0.850, indicating that the scales had good reliability and convergence validity.

To assess discriminant validity, two methods were used. First, Fornell–Larcker criteria were used [81]. This criteria indicates that when the square root of AVE is larger than the correlation coefficient of each latent variable, each latent variable has discriminant validity. Secondly, the cross loadings of the measured items must be checked. Second, to test the cross-loading of the measured items, the method must verify that each parameter has a larger factorial load in its own construction than in other constructions.

A discriminant validity evaluation based on Fornell–Larcker criterion is shown in Table 7. The bold and slanted diagonal values are the square root of AVE of each component, while the other values are the correlations amongst component. It can be seen that the values on the diagonal are all larger than those on the horizontal or vertical columns, which provides the first evidence of discriminating validity.

Further evidence for discriminating validity was provided by examining the cross-loading of items measured. Table 8 shows that each measurement item had the highest loading in its corresponding structure and there was no cross-loading problem, indicating that there was no multicollinearity problem between items loaded by different components in the outer measurement model [83]. Therefore, the measurement model established in this study was reliable and effective.

#### 3.4.2. Evaluation of Inner Structure Model

**Figure 2:**
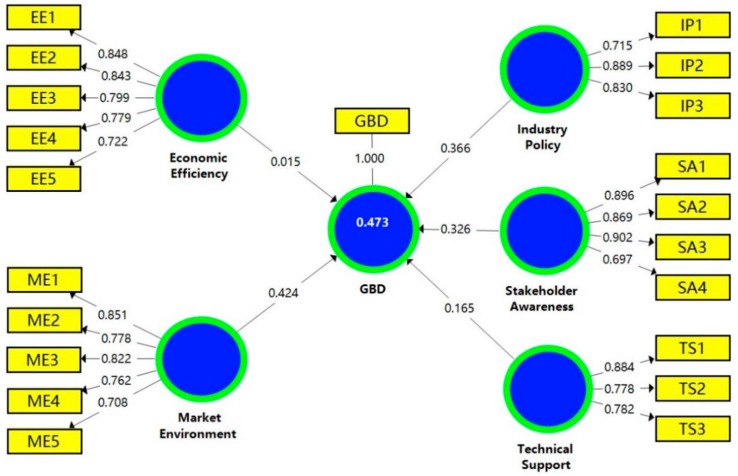
Assessment results of path coefficients.

After verifying the reliability and validity of the measurement model, in order to test the assumed path in the structural model, the path coefficient had to be evaluated. Path coefficients represent the assumptions that connect components [84]. Therefore, bootstrapping was developed and used as a general technique. The assessment results of the path coefficient are shown in Figure 2. In addition, Figure 2 also shows that the determination coefficient (R2) of GBD potential barriers was 0.473, which is greater than the threshold value of 0.200 [85]. Considering the influence of GBD potential barriers to diversity, the structure of the R2 value was satisfactory.

Table 9 shows the bootstrapping results for the structural model. The critical value test standard of double-tail test was: *t* > 1.65 means weak significance (10%); *t* > 1.96 means significant (5%); and *t* > 2.58 means extremely significant (1%). Rampasso et al. [86] suggested that *t*-values below 1.96 are not supported. Therefore, the path “P1: EE→GBD” and the path “P5: TS→GBD” were not supported, indicating that economic efficiency and technical support had no significant impact on GBD. In addition, the *t*-values of path “P2: IP→GBD”, path “P3: ME→GBD” and path “P4: SA→GBD” were all greater than 2.58, and the *p*-values were statistically significant at the confidence level of 1%, so these three paths were supported.

## 4. Discussion

According to the revealed results, it can be found that the respondents generally believed that the immature market environment is a crucial barrier to GBD in China. The coefficient of “P3: ME→GBD” reached 0.424, and the *t*-value reached 3.304, indicating that the market environment has a significant impact on GBD. This problem is very common in developing countries because the GBD level is relatively sluggish and the market is still in the exploratory stage [87]. In addition, due to the lack of training, education and promotion of green building, it is difficult for stakeholders to obtain effective knowledge concerning green building [88]. In fact, the training and promotion of green building can have a geometric effect on the market demand [89]. In addition, green building demonstration projects are critical to accelerating the development of green buildings, as they help to demonstrate the effectiveness of green buildings to the public.

Another important barrier concerns industry policies. As the results showed, the coefficient of path “P2: IP→GBD” reached 0.366, and the *t*-value reached 3.053. In other words, a lack of policy and industry guidance is one of the most important barriers for GBD in China. In China, the supervision of green buildings involves many governmental departments, such the housing and construction departments, environmental protection departments, and other governmental functional agencies. Because the current policies are relatively general, various departments and agencies have not been closely linked, leading to a phenomenon of prevarication or overlapping responsibilities among different departments [90]. Thus, the supervision system of green buildings needs to be improved.

The coefficient of path “P4: SA→GBD” reached 0.326, and the *t*-value reached 2.880, which proved that the environmental awareness of different stakeholders has a significant impact on GBD. In addition to the supervision systems, governments can also promulgate incentives to improve the stakeholders’ awareness, thus promoting green building development. Chan and Yung [91] believed that the government can use incentive tools to promote the development of green buildings. However, although the central and local governments have introduced incentive policies (e.g., subsidies for green building certified projects) to promote the development of green building, the effect has not been obvious. The reason is that under the joint effect of the rigid demand of real estate and the gap of green building knowledge, the awareness of “green” among stakeholders is weak [92]. However, in the more developed first-tier cities, people’s awareness of the importance of green building has been increasing, and many developers and contractors are choosing green and sustainable buildings [7].

Based on the statistical analysis results, the paths of EE→GBD and TS→GBD were not supported. It is noteworthy that the economic efficiency and the technological support were not recognized as the main factors affecting the development of green buildings in China. As an expert mentioned, China has already has advanced construction technologies and materials; however, these advanced green technologies and materials are not mainstream in the current construction market and need a strong cash flow support, which makes them unsuitable for large-scale popularization. Another expert also pointed out that GBD in China does not lack economic and technical feasibility, but it does lack the construction and management of the secondary market. As a result, the green building business into the mainstream of the construction market requires the government’s policy support. In addition, in order to promote the development of green building at the business scale, it is suggested that increasing the training and education of green building, building some green building demonstration projects, and enhancing the consciousness of stakeholders on green building and cognition should be the future directions of promoting GBD in China.

## 5. Conclusions

It is an emerging trend to build a more sustainable society, and green building can contribute to this target. The concept of green building has been promoted for decades in China, but the development status has not been optimistic so far. Thus, this paper aims to investigate the barriers encountered in the process of green building development in China. Through a systematic review of the existing research, 24 potential barriers of GBD were identified. After the EFA, a total of 20 barriers remained with a total interpretation variance of 66.057%. The remained barriers were divided into five components: Low economic efficiency, poor market environment, weak environmental awareness of stakeholders, imperfect industrial policies, and lack of relevant technical support.

The estimation results showed that the *t*-value of the path “P1: EE→GBD” and the path “P5: TS→GBD” were less than 1.96, indicating that economic efficiency and technical support had no significant impacts on GBD, so these two assumptions could not be supported. The *t*-values of paths “P2: IP→GBD”, “P3: ME→GBD”, and “P4: SA→GBD” were 3.053, 3.304, and 2.880, respectively, and the *p*-values had statistical significance at a 1% confidence level. Thus, the three paths were supported. Therefore, the research results showed that the lack of policy and industry guidance, immature market environment, and insufficient environmental awareness of different stakeholders are the main GBD barriers in China.

The results of this study can enable decision-makers to develop appropriate GBD strategies. Based on the revealed results, it is suggested that the government can further promote green building development by strengthening the supervision and implementation of green building, actively advocating circular economic theory, and combining these with necessary incentive measures. In addition, by enhancing training and education and by creating green building demonstration projects with public credibility, the public’s awareness can be improved.

## Figures and Tables

**Table 1:** Potential barriers of green building development.

Code	Barriers	Key Reference									
		[62]	[63]	[64]	[65]	[66]	[67]	[68]	[69]	[70]	[71]
B01	Lack of regulations and policy		**✓**			**✓**	**✓**		**✓**	**✓**	**✓**
B02	Lack of industrial guidance					**✓**					
B03	Lack of effective green building development modes		**✓**			**✓**				**✓**	
B04	Lack of effective supervision from government			**✓**	**✓**					**✓**	
B05	Inadequate support from related green building institutions			**✓**			**✓**	**✓**			**✓**
B06	Low level of green design					**✓**			**✓**		
B07	Incensement of construction cost		**✓**					**✓**	**✓**	**✓**	**✓**
B08	Extension of construction period		**✓**		**✓**				**✓**		
B09	Potential damage to structure					**✓**		**✓**		**✓**	
B10	Lack of mature green technology	**✓**			**✓**		**✓**	**✓**			**✓**
B11	Detachment of green building theories and technologies				**✓**	**✓**				**✓**	**✓**
B12	Higher price of green building	**✓**	**✓**	**✓**		**✓**					**✓**
B13	Economic benefit is not obvious in a short term	**✓**	**✓**					**✓**	**✓**		
B14	Lack of financial support	**✓**	**✓**	**✓**		**✓**	**✓**			**✓**	
B15	Immature green material market		**✓**		**✓**						**✓**
B16	Public concerns on quality of green materials		**✓**			**✓**				**✓**	
B17	Lack of environmental awareness from developer	**✓**					**✓**			**✓**	
B18	Lack of environmental awareness from contractor	**✓**					**✓**			**✓**	
B19	Lack of environmental awareness from public			**✓**			**✓**	**✓**		**✓**	
B20	Low demand for green buildings		**✓**		**✓**		**✓**	**✓**			**✓**
B21	Ineffectiveness effect of demonstration green building				**✓**	**✓**		**✓**			
B22	Lack of green construction training	**✓**	**✓**	**✓**					**✓**		**✓**
B23	Lack of publicity for green buildings	**✓**		**✓**						**✓**	
B24	Limited benefit to enterprise reputation	**✓**				**✓**			**✓**		

**Table 2:** Personal background information of the respondents.

Variable	Category	Frequency	Percentage (%)
Workplace	Developer	21	26.92
	Contractor	16	20.51
	Government	13	16.67
	Scholar	28	35.90
Working experience (year)	0–5	60	76.92
	6–10	12	15.38
	11–15	3	3.85
	Above 15	3	3.85
Education level	PhD	6	7.69
	Master	43	55.13
	Bachelor	27	34.62
	Others	2	2.56
Number of on-going project workers	1–50	31	39.74
	51–100	16	20.51
	101–200	13	16.67
	Above 200	18	23.08

**Table 3:** Ranking of the potential barriers.

Rank	Code	Barriers	Mean	SD ^1^
1	B04	Lack of effective supervision from government	4.0769	0.99046
2	B17	Lack of environmental awareness from developer	4.0128	0.99992
3	B15	Immature green material market	4.0128	1.02556
4	B02	Lack of industrial guidance	3.9872	0.99992
5	B07	Incensement of construction cost	3.9615	1.21080
6	B14	Lack of financial support	3.9231	1.09033
7	B05	Inadequate support from related green building institutions	3.8974	1.07619
8	B03	Lack of effective green building development modes	3.8718	0.94469
9	B01	Lack of regulations and policy	3.8718	1.18824
10	B10	Lack of mature green technology	3.8205	1.06593
11	B21	Ineffectiveness effect of demonstration green building	3.8077	0.94054
12	B24	Limited benefit to enterprise reputation	3.7692	1.09216
13	B08	Extension of construction period	3.7436	1.11000
14	B12	Higher price of green building	3.7308	1.07719
15	B11	Detachment of green building theories and technologies	3.7179	0.97897
16	B13	Economic benefit is not obvious in a short term	3.5769	1.12260
17	B20	Low demand for green buildings	3.5769	1.26408
18	B19	Lack of environmental awareness from public	3.5641	1.19076
19	B06	Low level of green design	3.5513	1.02751
20	B22	Lack of green construction training	3.5000	1.02881
21	B18	Lack of environmental awareness from contractor	3.4872	1.20328
22	B23	Lack of publicity for green buildings	3.4744	1.01578
23	B16	Public concerns on quality of green materials	3.0769	1.15959
24	B09	Potential damage to structure	2.7949	1.22059

^1^ SD: Standard Deviation.

**Table 4:** ANOVA for the five background variables.

Code	Workplace		Experience		Gender		Education		Number of Workers	
	F	Sig.	F	Sig.	F	Sig.	F	Sig.	F	Sig.
B01	0.557	0.645	0.890	0.451	1.503	0.224	2.053	0.114	2.322	0.082
B02	0.806	0.495	1.837	0.148	0.010	0.921	1.107	0.352	2.562	0.061
B03	0.620	0.604	0.366	0.778	0.606	0.439	3.356	**0.023**	0.546	0.652
B04	1.768	0.161	1.975	0.125	0.323	0.571	0.789	0.504	1.044	0.378
B05	1.772	0.160	0.545	0.653	0.276	0.601	2.727	**0.050**	1.743	0.166
B06	2.096	0.108	0.824	0.485	0.369	0.546	1.763	0.162	1.159	0.331
B07	1.145	0.337	1.236	0.303	0.060	0.807	2.516	0.065	0.299	0.826
B08	1.943	0.130	1.529	0.214	0.310	0.579	1.745	0.165	1.168	0.328
B09	0.318	0.813	1.615	0.193	0.550	0.461	0.191	0.902	1.651	0.185
B10	0.582	0.629	2.906	**0.040**	0.055	0.815	1.014	0.391	1.327	0.272
B11	1.760	0.162	0.417	0.741	0.107	0.745	0.991	0.402	1.294	0.283
B12	3.083	**0.032**	1.005	0.396	3.068	0.084	1.446	0.236	1.511	0.219
B13	0.746	0.528	0.211	0.888	0.126	0.723	1.962	0.127	0.352	0.788
B14	1.218	0.309	0.301	0.824	4.225	**0.043**	0.588	0.625	2.525	0.064
B15	0.667	0.575	1.107	0.352	0.656	0.420	1.620	0.192	1.367	0.259
B16	1.117	0.348	3.419	**0.022**	0.441	0.509	3.511	**0.019**	0.468	0.706
B17	3.919	**0.012**	1.450	0.235	6.826	**0.011**	3.074	**0.033**	0.331	0.803
B18	2.414	0.073	1.561	0.206	3.302	0.073	2.614	0.057	0.583	0.628
B19	2.276	0.087	0.323	0.808	1.727	0.193	1.026	0.386	0.849	0.471
B20	2.338	0.080	0.646	0.588	1.336	0.251	1.155	0.333	0.579	0.631
B21	3.244	**0.027**	2.152	0.101	1.217	0.273	0.288	0.834	1.735	0.167
B22	2.088	0.109	1.721	0.170	0.049	0.826	0.643	0.590	1.990	0.123
B23	1.858	0.144	1.463	0.232	4.139	**0.045**	0.327	0.806	1.780	0.158
B24	2.407	0.074	2.856	**0.043**	0.001	0.975	0.685	0.564	2.908	**0.040**

**Table 5:** Factor analysis result of the selected potential barriers.

Code	Rotated Component Matrix ^1^					New Code
	Component					
	1	2	3	4	5	
B07	0.759	-	-	-	-	EE1
B13	0.759	-	-	-	-	EE2
B08	0.720	-	-	-	-	EE3
B12	0.681	-	-	-	-	EE4
B14	0.609	-	-	-	-	EE5
B22	-	0.745	-	-	-	ME1
B23	-	0.742	-	-	-	ME2
B21	-	0.702	-	-	-	ME3
B24	-	0.630	-	-	-	ME4
B15	-	0.540	-	-	-	ME5
B18	-	-	0.885	-	-	SA1
B17	-	-	0.857	-	-	SA2
B19	-	-	0.838	-	-	SA3
B20	-	-	0.507	-	-	SA4
B04	-	-	-	0.776	-	IP1
B02	-	-	-	0.768	-	IP2
B01	-	-	-	0.707	-	IP3
B05	-	-	-	-	0.728	TS1
B06	-	-	-	-	0.633	TS2
B03	-	-	-	-	0.544	TS3
**Eigenvalues**	3.715	3.477	3.285	2.896	2.483	-
**Variance (%)**	15.479	14.489	13.686	12.058	10.345	-
**Cumulative (%)**	15.479	29.968	43.654	55.712	66.057	-

^1^ Note: Rotation converged in eight iterations; KMO measure of sampling adequacy: 0.814; Bartlett’s test of sphericity: Approximate chi-square 1129.561, degree of freedom (df) 276, significance (sig.) 0.000.

**Table 6:** Assessment results of reliability analysis and convergent validity.

Component	Code	Loading	Cronbach’s α	AVE	CR
Economic Efficiency	EE1	0.848	0.858	0.639	0.898
	EE2	0.843	-	-	-
	EE3	0.799	-	-	-
	EE4	0.779	-	-	-
	EE5	0.722	-	-	-
Market Environment	ME1	0.851	0.844	0.617	0.889
	ME2	0.778	-	-	-
	ME3	0.822	-	-	-
	ME4	0.762	-	-	-
	ME5	0.708	-	-	-
Stakeholder Awareness	SA1	0.896	0.863	0.715	0.908
	SA2	0.869	-	-	-
	SA3	0.902	-	-	-
	SA4	0.697	-	-	-
Technical Support	TS1	0.859	0.747	0.666	0.856
	TS2	0.800	-	-	-
	TS3	0.739	-	-	-
Industry Policy	IP1	0.715	0.741	0.663	0.854
	IP2	0.889	-	-	-
	IP3	0.830	-	-	-

**Table 7:** Discriminant validity evaluation based on Fornell–Larcker criteria.

Component	EE	IP	ME	GBD	SA	TS
**EE**	***0.800*** ^1^	-	-	-	-	-
**IP**	0.580	***0.814*** ^1^	-	-	-	-
**ME**	0.598	0.596	***0.786*** ^1^	-	-	-
**GBD**	0.381	0.428	0.610	***1.000*** ^1^	-	-
**SA**	0.397	0.278	0.503	0.549	***0.845*** ^1^	-
**TS**	0.652	0.684	0.559	0.287	0.258	***0.816*** ^1^

^1^ The square root of AVE for each component.

**Table 8:** Cross loadings of the hypothesized model.

Code	EE	IP	ME	SA	TS
**EE1**	**0.848** ^1^	0.531	0.399	0.220	0.578
**EE2**	**0.843** ^1^	0.514	0.524	0.415	0.570
**EE3**	**0.799** ^1^	0.355	0.409	0.205	0.489
**EE4**	**0.779** ^1^	0.416	0.459	0.417	0.454
**EE5**	**0.722** ^1^	0.471	0.569	0.317	0.495
**IP1**	0.509	**0.715** ^1^	0.479	0.308	0.676
**IP2**	0.408	**0.889** ^1^	0.526	0.177	0.536
**IP3**	0.505	**0.830** ^1^	0.446	0.199	0.462
**ME1**	0.385	0.480	**0.851** ^1^	0.432	0.458
**ME2**	0.428	0.427	**0.778** ^1^	0.314	0.439
**ME3**	0.462	0.513	**0.822** ^1^	0.353	0.466
**ME4**	0.477	0.449	**0.762** ^1^	0.505	0.380
**ME5**	0.619	0.466	**0.708** ^1^	0.366	0.452
**SA1**	0.335	0.274	0.428	**0.896** ^1^	0.227
**SA2**	0.354	0.275	0.398	**0.869** ^1^	0.214
**SA3**	0.310	0.233	0.437	**0.902** ^1^	0.200
**SA4**	0.357	0.142	0.451	**0.697** ^1^	0.238
**TS1**	0.554	0.551	0.420	0.281	**0.884** ^1^
**TS2**	0.422	0.519	0.464	0.076	**0.778** ^1^
**TS3**	0.618	0.600	0.478	0.275	**0.782** ^1^

^1^ Bold values show that each measurement item had the highest loading on its respective construct.

**Table 9:** Evaluation results of the structural model.

No.	Path	Path Coefficient	*t*-Value	*p*-Value	Inference
P1	EE → GBD	0.015	0.140	0.889	Not supported
P2	IP → GBD	0.366	3.053	***	Supported
P3	ME → GBD	0.424	3.304	***	Supported
P4	SA → GBD	0.326	2.880	***	Supported
P5	TS → GBD	0.165	1.181	0.238	Not supported

*** Coefficient is statistically significant at the 1% level of confidence.
